# The Active Recovery Triad monitor: evaluation of a model fidelity scale for recovery-oriented care in long-term mental health care settings

**DOI:** 10.1186/s12888-022-03949-5

**Published:** 2022-05-19

**Authors:** Lieke Johanna Cornelia Zomer, Lisette van der Meer, Jaap van Weeghel, Anne Laura van Melle, Henrica Cornelia Wilhelmina de Vet, Martijn Kemper, Guy Antoine Marie Widdershoven, Yolande Voskes

**Affiliations:** 1 Department of Ethics, Law and Humanities, Amsterdam UMC location Vrije Universiteit Amsterdam, Amsterdam, the Netherlands; 2 Department of Clinical & Developmental Neuropsychology, University of Groningen, Groningen, the Netherlands; 3 Department of Rehabilitation, Lentis Psychiatric Institute, Zuidlaren, the Netherlands; 4 Tranzo Scientific Center for Care and Wellbeing, Tilburg University, Tilburg, the Netherlands; 5 Phrenos Center of Expertise On Severe Mental Illness, Utrecht, the Netherlands; 6 GGZ inGeest, Amsterdam, the Netherlands; 7 Department of Epidemiology and Data Science, Amsterdam UMC location Vrije Universiteit Amsterdam, Amsterdam, the Netherlands; 8 Vanuit de Grond, Haarlem, the Netherlands; 9 Impact Care Group, GGz Breburg, Tilburg, the Netherlands

**Keywords:** ART model, Model fidelity scale, Long-term mental health care, Psychometric properties

## Abstract

**Objective:**

The Active Recovery Triad (ART) model is a recently developed care model for people who are admitted to an institutional setting for several years and receive 24-h mental health care and support. This study focuses on the ART monitor, a model fidelity scale that measures the degree of compliance with the ART model. Our aim is to evaluate the psychometric properties of the ART monitor and to further improve the instrument.

**Methods:**

Fifteen teams at the start (*n* = 7, group 1) or in the process (6 months to three years) of implementing care according to the ART model (*n* = 8, group 2) were audited using the ART monitor. Auditors were trained care workers, peer workers, and family peer workers. Auditors and team members provided feedback on the instrument. The content validity, construct validity and inter-rater reliability of the ART monitor were investigated. Based on the outcomes of these psychometric properties, the ART monitor was finalized.

**Results:**

Regarding content validity, auditors and teams indicated that they perceived the ART monitor to be a useful instrument. In terms of construct validity, a significant difference (t(13) = 2.53, *p* < 0.05) was found between teams at the start of the implementation process (group 1, average score of 2.42 (SD = 0.44)) and teams with a longer duration of implementation (group 2, average score of 2.95 (SD = 0.37)). When allowing for a one-point difference in scores, 88% of the items had an inter-rater agreement over 65%. Items with a relatively low inter-rater reliability, in combination with feedback from auditors and teams regarding content validity, provided direction for further improvement and revision of the instrument.

**Conclusions:**

We concluded that the revised ART monitor is feasible and useful in mental health care practice. However, further evaluation of its psychometric properties will be needed.

**Supplementary Information:**

The online version contains supplementary material available at 10.1186/s12888-022-03949-5.

## Background

There have been several developments in the past decade aimed at improving the quality of mental health care for people with serious mental illnesses. Examples include care models such as (Flexible) Assertive Community Treatment (F-ACT), Safewards, Intensive Home Treatment (IHT) and High and Intensive Care (HIC) [1–5]. However, there is one group of service users in mental health care who have not benefitted much from these developments, namely people who are admitted to an institutional setting for several years and receive 24-h care and support. These individuals have various needs in multiple life domains and often live in isolation with no or few family contacts [6–8]. A newly developed care model in the Netherlands, the Active Recovery Triad (ART) model, aims to alter the prospects of these service users and contribute to the quality of care by fostering their recovery process and not regarding them as permanent residents of long-stay facilities [9, 10]. The ART model is an integrated care model focusing on four dimensions of *Recovery* (recovery of health, personal identity, daily life and community functioning), as well as the *Active* attitude of service users, significant others, and care professionals, the latter three collectively referred to as the *Triad* (for further explanation of the ART model, see Additional File 1).

The ART model was developed by multiple stakeholders in the field of long-term mental health care [9, 10]. The principles of the ART model were defined based on scientific evidence, the practical experience of the involved professionals, and the experiences of (ex) service users, family and significant others. Because of this collaborative process, the ART model quickly gained a great deal of attention throughout the country [10].

In order to assess to what extent the ART model is implemented as intended within teams, also referred to as implementation fidelity [11], the content of the ART model was operationalized into a model fidelity scale: the ART monitor. Model fidelity scales are common instruments for quality improvement in practice and for research purposes [12–14]. The literature shows that working with a model fidelity scale is beneficial for implementing a new care model into practice and for service users outcomes [12, 15, 16]. In addition, using such tools to support the implementation process or improve the quality of care are considered helpful, because implementing new routines requires substantial effort and behavioral changes which are often difficult to accomplish. There are other existing tools, comparable to the ART monitor, to evaluate the quality of recovery-oriented care, such as the Quality Indicator for Rehabilitative Care (QuIRC) and the Recovery Oriented Practices Index (ROPI. However, these are less comprehensive or come without a collaboratively developed vision or model [17, 18]. The collaboratively developed vision of the ART model extends the scope of other measurements. The ART monitor serves as a checklist to review a team’s degree of compliance with the ART model. By assessing the ART monitor themselves or by means of an audit, a team is able to monitor the implementation and receives feedback on how to proceed in this process. This monitoring and feedback facilitates the level of fidelity within teams [11].

The aim of the current study is to evaluate the psychometric properties of the ART monitor, as part of the iterative process to further develop the instrument in collaboration with Dutch mental health organizations. Three important and commonly used psychometric properties will be investigated, namely content validity, construct validity, and inter-rater reliability [19–23]. By examining and, where necessary, improving the quality of the instrument, its usefulness as a guiding set of standards for recovery-oriented care can be fostered. Also, insight into its psychometric properties is needed for research purposes, as the ART monitor can be used in follow-up studies, for example to investigate the relationship between ART model compliance and intended outcomes, such as quality of care or service user recovery outcomes.

## Methods

### Instrument

The first version of the ART monitor consisted of 51 items, divided into nine domains. The structure of the ART monitor was as follows, listed by domain of the instrument:

- 1) Team structure included items regarding the team composition in terms of disciplines.
- 2) Team process addressed items related to the competencies of care workers, e.g., attitude and how they collaborate.
- 3) Recovery-oriented care and support included items based on the seven steps of the ART model for structuring care and support and working on recovery (the seven steps are explained in Additional File 1 and Zomer et al. [10]).
- 4) Other principles of recovery-oriented care and support comprised items regarding the professional aspects of the long-term care setting, including knowledge of professional guidelines, diagnoses and medication.
- 5) Organization of care included important preconditions for the care process, e.g., admission and discharge, waiting list and consultation.
- 6) Professionalization focused on items regarding training, education and reflection of team members.
- 7) Healing environment addressed important preconditions regarding the housing of service users.
- 8) Safety captured items related to expertise on and dealing with safety and safety management.
- 9) Reduction of coercion focused on the evaluation of coercive measures.

The development process of the ART model and the ART monitor has been described in Additional File 1. Table 1 shows three examples of items in the ART monitor. Some of these items focused on structural components of the model, including items regarding the professional disciplines in a team, such as “Peer worker and family peer worker”. In addition, effort has been made to incorporate items that are process oriented, for example, “Cooperation in the triad” and “Team spirit”. Scoring of the ART monitor was performed through an audit. The items were scored on a five-point scale, with response options ranging from 1 (not compliant) to 5 (fully compliant). However, for some items, fewer response options were available, depending on the content of the items or the number of criteria. An example is the item “Cooperation in the triad”, as depicted in Table 1. For this item and fifteen others, only scores 1, 3, and 5 were available; for one item, scores 1, 2, 4, and 5 were available.

**Table 1:** Examples of items in the ART monitor

Item	Score 1	Score 2	Score 3	Score 4	Score 5
*Peer workers and family peer workers* In total, there are 2 FTE peer workers and family peer workers available	There are 0.49 or fewer FTE (family) peer workers available	There are from 0.5 to 0.99 FTE (family) peer workers available	There are from 1 to 1.49 FTE (family) peer workers available	There are from 1.5 to 1.99 FTE (family) peer workers available	There are 2 or more FTE (family) peer workers available
*Community participation* •All service users are being supported with a form of community participation •Occupational therapists and the rest of the team work towards increasing the community participation of service users •Recovery colleges are being introduced to service users •The team acquires and maintains contact with organizations in the community •The team has contact with municipalities on a regular basis regarding community participation •Rehabilitation interventions and individual placement and support (IPS) are used to stimulate community participation	The team meets none of the criteria	The team meets one criterion	The team meets two or three criteria	The team meets four or five criteria	The team meets all six criteria
*Cooperation in the triad* •At the individual level, there is cooperation between service users, their significant others, and care workers •At the team level, service users and significant others are involved in change processes and their feedback is requested on a regular basis •At the organizational level, service users and significant others are involved in policy making	The team meets none of the criteria	-	Aspects of the three criteria are recognizable. However, there is no consistency regarding the cooperation in the triad	-	The team meets all three criteria

### Participants

Twenty Dutch mental health care organizations agreed to participate in the research project. Five organizations were in the orientation phase and did not actively want to start the implementation process of the ART model. Therefore, they were not ready to participate in the data collection, but they committed themselves to the ART model and provided financial support to complete this study. The other fifteen organizations actively participated in the data collection. Participation entailed the selection of one team to be audited and a feedback meeting using the ART monitor. Additionally, in every organization, a central contact person was appointed for communication related to and the organization of the data collection on location, and two or three persons were selected to become auditors.

A convenience sampling method was used, in which all fifteen organizations chose one team to participate in this study [24]. The selection of teams was performed by the central contact person of each organization, in agreement with the management and the team members concerned. There were only two important inclusion criteria, namely teams had to provide long-term psychiatric care for people with serious mental illnesses and either were at the start or in the process of implementing care according to the ART model. As a result, the degree of compliance with the ART model varied among the included teams. Two groups were identified. The first group consisted of seven organizations that chose to select a team at the start of the implementation process, allowing to use the results of the ART monitor as a baseline measurement to actively start the implementation process (group 1). These teams were already familiar with the ART model, but they had not structurally started to work according to this model. The other eight organizations chose to select a team that was further along in the implementation process, allowing to use the ART monitor results to provide insights to set new goals for future implementation (group 2). All teams followed their own course of implementation, which allowed each of them to prioritize certain elements of the ART model initially. Implementation was fostered by suggestions from the ART handbook, the ART monitor, national conferences, platform meetings and other small symposia regarding ART to stimulate knowledge sharing and experiences among teams and organizations [9]. Of the fifteen teams that participated in this study, eight were situated at open long-stay wards, two at partially open/partially closed long-stay wards, one at a closed long-stay ward and four at housing facilities situated at an institution.

### Auditing process

#### Selection of auditors

The selection of auditors, who visited another organization to perform an audit using the ART monitor, was assigned to the central contact person of every participating organization. Inclusion criteria for the auditors were as follows: 1) basic knowledge of the ART model, 2) support for this vision and 3) be prepared to review an organization other than their own. People who volunteered to become auditors often did so because they were eager to learn from other organizations. Frequently, they already played an important role in the implementation of the ART model within their own organization or team, for example, working as a project leader, on a team implementing the model, or in a consulting role from the perspective of peer worker or family peer worker. The central contact person was instructed to assess the ability of the persons to perform audits, in consultation with the researchers (LZ and YV). They were explicitly asked to make an effort to recruit auditors not only from among mental health professionals but also peer workers and/or family peer workers.

All involved organizations, except two, provided auditors. In total, these audits were performed by 26 auditors: three peer workers, four family peer workers, five nurses, four social workers, four nurse practitioners, three managers, one psychiatrist, and two mental health specialists not otherwise specified. The auditors varied in years of work experience in care practice. Three auditors already had experience conducting audits for the HIC study [25].

#### Training of auditors

All 26 auditors participated in a one-day training program. This training was led by the main researcher (LZ), researchers with experience based on similar procedures in acute psychiatry with the HIC monitor (YV and LvM), and an experienced HIC auditor (MK) [25]. During this training, the principles of performing an audit, conducting interviews, and scoring the ART monitor were explained and practiced. Specific attention was placed on confidentiality of the information discussed during audits. All auditors agreed to the confidentiality terms by the performance of an audit. During the course of the study, four follow-up meetings were organized to maintain and foster the skills of the auditors. In addition, during these meetings, auditors were actively asked to provide feedback on the ART monitor, which was used as data to investigate the quality of the instrument.

#### Audits

Data were collected by means of audits using the ART monitor. The initial plan was to perform the audits with three auditors (two ‘regular’ care workers and a peer worker or family peer worker). Due to challenges in the planning process, a shortage of (family) peer workers, and issues arising from auditors who quit prior to the end of the study (for example, because they obtained a new job), five audits were performed by only two auditors with ‘regular’ care backgrounds. The other audits were performed by three auditors, according to the initial plan. Prior to the audit, auditors received background information about the team (e.g., different disciplines, caseload, housing conditions, number of vacancies). Audits lasted one day (eight hours). The auditors received a tour of the facility, attended a multidisciplinary meeting, performed a group interview with a selection of team members (including at least one person from every discipline), and conducted an interview with two service users and their significant others. At the end of the day, the auditors examined four anonymized clinical records in terms of their consistency with the content of the interviews. Prior to this file check, the service users granted permission by means of informed consent. Based on this aggregated information, the auditors scored the ART monitor independently from each other.

Four pilot audits were performed to check the content of the ART monitor (relevance, comprehensiveness, and comprehensibility) and the audit procedure. Small adaptations were made in the content of the ART monitor (e.g., correction of typing errors, clarifications of concepts, and small adaptations in the items on housing conditions and the evaluation of coercive measures). The auditors, teams and researchers considered the audit procedure to be of a quality appropriate to the continuation of the study.

#### Feedback meetings

Two weeks after an audit, the main researcher (LZ) held a group meeting with the teams. During this meeting, the scores on the ART monitor were discussed to provide the teams with insight into the degree of compliance with the ART model. The opinions of the team members regarding their performance on the ART monitor, as well as their feedback on the form and content of this monitor, were used as input to assess the content validity of the instrument.

### Analysis

The analysis of this study focused on the evaluation of the psychometric properties of the ART monitor. Based on the outcomes of this evaluation, the ART monitor was finalized.

#### Investigating the psychometric properties

In order to evaluate the psychometric properties of the ART monitor, content validity, construct validity, and inter-rater reliability were investigated. Particularly in this early stage of working with this new instrument, these psychometric properties provided fruitful input for further improvement of the ART monitor [13, 22]. We were specifically interested in scores on individual items, as teams take individual items into account when improving their care and support. Since the goal of our study was not to reduce the items or present sub scores for domains, but rather allow the ART monitor to serve as a checklist to improve the quality of care, factor analysis and internal consistency were not considered.

#### Content validity

The evaluation of content validity consisted of two steps. First, qualitative feedback was gathered. The relevance, comprehensiveness and comprehensibility were examined from the perspective of the assessors (auditors) and the teams that use the ART monitor in practice as a tool to implement the ART model [22, 26]. The feedback was gathered at three moments: 1) from auditors after every audit, 2) during the follow-up meetings with auditors and 3) during feedback meetings with the teams. Emphasis was placed on the relevance of the items, scoring options, comprehensiveness of the instrument, topics that were missed, and comprehensibility and clarity of the concepts in the ART monitor. The feedback was sorted and analyzed for each item. The second step was to compare the average scores and standard deviation (SD) of individual items [20]. Structurally low or high scores for specific items might imply that they are not distinctive enough, that they might contain elements that are either too easy or too difficult to implement, or that some elements are already common in mental health practice while others are more novel. These qualitative and quantitative data provided input about if and where to make changes to finalize the ART monitor.

#### Construct validity

To investigate construct validity, also referred to as discriminative validity or known group validity, it was examined whether the outcomes of the measurements were consistent with the hypothesis that a longer implementation process leads to higher scores on the ART monitor [12, 19, 22]. Mokkink et al. [22] referred to this type of construct validity as “hypothesis testing”. Although there are similarities between, for example, the QuIRC or ROPI and the ART monitor, the ART monitor is more comprehensive, which limits the comparability of the monitor to these other measures. Our hypothesis was that teams that have been in the process of implementing care according to the ART model longer are likely to achieve higher scores on the ART monitor compared to teams at the start of the implementation process. To test this hypothesis, the variability between teams related to their stage of implementation was used and the audit scores of group 1 were compared with the audit scores of group 2. For group 1 (*n* = 7), the audit was the starting point for their implementation process. For group 2 (*n* = 8), implementation of the ART model was fostered by a longer involvement with national ART conferences, platform meetings and other symposia, internal working groups, and a longer time to familiarize themselves with the ART model by means of the ART handbook and the ART monitor. For each team, the average score on the ART monitor was calculated by the sum of all scores, divided by the total number of items. Since all items include a scoring range from 1 to 5, the differences in scoring options had no effect on calculating the average scores. In fact, an average reflects a score that is simple to interpret for care workers and it allows us to compare outcomes of the current study with future audit scores. An independent t-test was performed to test our hypothesis by comparing the average scores of the two groups on the ART monitor.

#### Inter-rater reliability

In order to assess the inter-rater reliability, differences in scores between auditors were examined [23]. For each item, the percentage of the corresponding scores was calculated. This is an absolute measure (a measure of agreement) and more informative compared to other measures of inter-rater reliability such as Cohen’s Kappa or the intraclass correlation coefficient (ICC) (relative measures, measures of reliability) [27]. This measure does not include chance agreement, which is also not taken into account in care practice, and is not influenced by variety in scores between participating teams. Also, inter-rater reliability allowing a one-point difference in score was calculated [21]. Cut-off points of 85% (very good), 75% (good), and 65% (acceptable) were used for inter-rater reliability. These were arbitrary cut-off points that served as signs of the need to improve an item. Comparing results of exact agreement and allowing for a one-point difference provided extra information regarding auditors’ scoring of the items and items that may need improvement.

At the first auditor follow-up meeting, we noticed a different scoring strategy and focus between peer workers and family peer workers on the one hand and auditors with a ‘regular’ care background on the other hand on some items in the ART monitor (i.e., the nuances and weighting of some criteria differed). Peer worker and family peer worker auditors indicated that they were not familiar with reading and evaluating clinical records, which made the file check during the audit a challenge and might have affected their scores. Although this different focus may in fact be beneficial for practical use of the ART monitor, for the purpose of evaluating its psychometric properties, this is unfavorable. Therefore, in combination with the issues in the planning of the audits and the shortage of (family) peer workers, only the scores of the auditors with a ‘regular’ care background were included in the analysis, to ensure comparability of the interpretation of items and weighing of the criteria between auditors.

#### Finalizing the ART monitor

Based on the outcomes of the content validity and inter-rater reliability analyses, it was determined whether items of the ART monitor were in need of improvement in order to finalize the ART monitor. An overview was created of the following: 1) items with low inter-rater reliability (< 65% agreement); 2) items that received a lot of feedback regarding their relevance, comprehensiveness, and comprehensibility; and 3) items that scored structurally low or high. Items that met one or more of these criteria were critically reviewed by the researchers. When necessary, the feedback from auditors and teams regarding the relevance, comprehensiveness and comprehensibility provided direction on how to improve the items.

## Results

The aim of this study was to evaluate the psychometric properties of the ART monitor and further develop the instrument. This was done in close collaboration with teams from different mental health care organizations in the Netherlands, by means of fifteen audits and feedback meetings. First, the findings of the evaluation of the psychometric properties, including the content validity, construct validity, and inter-rater reliability, are presented. Then, the improvements made to the ART monitor, based on the outcomes of the evaluation, are described.

### Investigating the psychometric properties

#### Content validity

Regarding the content validity, auditors and teams indicated that they perceived the ART monitor as a useful instrument, but their feedback included several suggestions for improvement related to the relevance, comprehensiveness and comprehensibility of the ART monitor. First, the items with only scoring options 1, 3, or 5 seemed to lack distinctive capability. Second, some items were similar in terms of their content. Third, some principles of the ART model were not well represented in the model fidelity scale. Fourth, some items were perceived as (partially) unclear. Fifth, some items did not fit into all settings where the ART model is being implemented (e.g., different standards are needed for (closed) clinical wards versus housing facilities). Sixth, some domain titles were unclear (e.g., the domain “Other principles of recovery-oriented care and support”).

In addition, Table 2 shows the average score on all individual items of the ART monitor. The average score on all items was 2.70. High scoring items were “Safety management system”, “Psychiatrist”, “Somatic care”, “Reachability”, and “Conflict control and personal safety”. The five lowest scoring items were “Cooperation with FACT and other outpatient care teams”, “Peer worker and family peer worker”, “Occupational therapist”, “Regional teams” and “Dual diagnosis”. Half the structurally high- or low-scoring items had only scoring options of 1, 3, or 5, which indicates that these items might not be distinctive enough.

**Table 2:** Average score and inter-rater reliability per item of the ART monitor (*N* = 15)

Item	Average score (SD)	Exact Agreement	Agreement allowing 1-point difference
**Team structure**			
1. Caseload^a^	3.27 (1.64)	66.7%	-
2. Team composition	2.77 (1.14)	26.7%	86.7%
3. Peer worker and family peer worker	1.37 (0.67)	80.0%	100.0%
4. Nurses	3.87 (1.14)	53.3%	66.7%
5. Nurse practitioner	2.70 (1.66)	53.3%	86.7%
6. Social workers/residential support worker	3.27 (1.80)	73.3%	100.0%
7. Occupational therapist	1.37 (0.77)	86.7%	93.3%
8. Psychiatrist^b^	3.87 (1.39)	73.3%	100.0%
9. Health care psychologist/behavioral specialist	2.97 (1.85)	73.3%	80.0%
10. Extra disciplines	3.17 (1.05)	53.3%	100.0%
**Team process**			
11. Vision and working method^a^	2.53 (1.01)	66.7%	-
12. Community participation	2.27 (1.08)	33.3%	86.7%
13. Hospitality and presence	3.43 (0.73)	46.7%	93.3%
14. Attitude of staff	2.60 (0.81)	60.0%	86.7%
15. Active recovery^a^	2.53 (0.86)	66.7%	-
16. Working in the triad^a^	3.07 (0.37)	93.3%	-
**Recovery-oriented care and support**			
17. Intake	2.23 (0.84)	26.7%	73.3%
18. Care coordination meeting (CCM)	2.40 (1.19)	46.7%	86.7%
19. Revitalize or build resource group	2.33 (0.84)	33.3%	73.3%
20. Introduce recovery^a^	2.67 (1.06)	66.7%	-
21. Needs, strengths, and wishes	2.73 (1.02)	46.7%	80.0%
22. Integrated treatment and recovery plan	3.00 (0.91)	26.7%	80.0%
23. Recovery interventions at four levels^a^	2.40 (1.30)	80.0%	-
24. Systematic risk assessment	2.07 (1.11)	20.0%	80.0%
25. Early warning sign plan^a^	3.03 (1.13)	46.7%	-
26. Digital whiteboard	1.77 (1.31)	80.0%	93.3%
27. Rooming in^a^	2.20 (1.13)	73.3%	-
28. Stepped care	2.87 (1.07)	33.3%	86.7%
29. Recovery assessment^a^	2.13 (1.36)	66.7%	-
**Other principles of recovery-oriented care and support**			
30. Mental health care standards	2.37 (0.81)	46.7%	73.3%
31. Somatic care^a^	3.87 (1.14)	53.3%	-
32. Medication policy	3.80 (0.81)	20.0%	86.7%
33. Dual diagnosis	1.73 (0.91)	60.0%	80.0%
**Organization of care**			
34. Cooperation with FACT and other outpatient care teams^a^	1.27 (0.87)	80.0%	-
35. Admission and discharge	2.37 (1.19)	40.0%	73.3%
36. Care process and consultation	2.27 (1.53)	73.3%	80.0%
37. Waiting list	2.47 (1.50)	53.3%	80.0%
38. Reachability	4.03 (1.19)	26.7%	73.3%
39. Regional teams^a^	1.63 (0.93)	73.3%	-
40. ART-improvement curve	2.17 (1.44)	60.0%	80.0%
**Professionalization**			
41. Reflection^a^	2.13 (1.46)	66.7%	-
42. Training and education	2.13 (0.86)	26.7%	60.0%
43. Knowledge of regional network^a^	2.60 (1.22)	66.7%	-
44. Team spirit	3.40 (0.93)	46.7%	86.7%
**Healing environment**			
45. Healthy living environment	1.93 (1.02)	53.3%	93.3%
46. Housing first^a^	1.77 (1.28)	46.7%	-
47. Housing conditions	2.63 (1.00)	26.7%	80.0%
**Safety**			
48. Safety management system	4.47 (0.73)	60.0%	86.7%
49. Conflict management and personal safety^a^	4.20 (1.35)	60.0%	-
50. Cooperation agreements on safety	3.23 (1.38)	40.0%	80.0%
**Reduction of coercion**			
51. Evaluation of coercive measures	3.03 (1.50)	33.3%	53.3%

^a^item with scoring options 1, 3, and 5

^b^item with scoring options 1, 2, 4, and 5

#### Construct validity

**Fig. 1:**
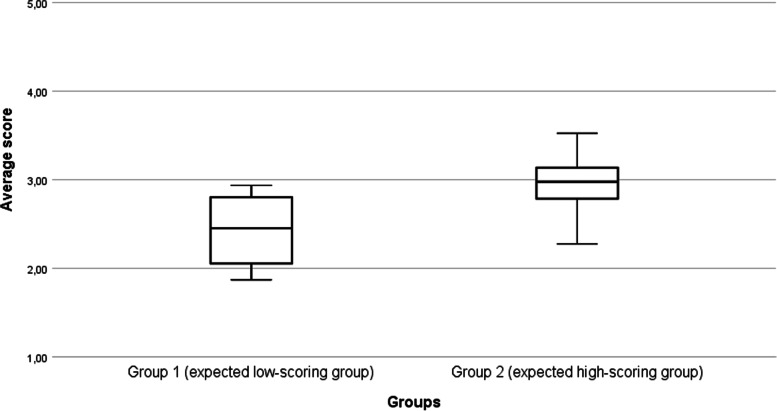
Scores of group 1 (expected low-scoring group) and group 2 (expected high-scoring group)

In order to assess the construct validity, two groups of teams were compared. For group 1 (*n* = 7, the expected low-scoring group), the audit was the starting point of the implementation process. Within group 2 (*n* = 8, expected high-scoring group), the duration of implementation varied between six months and three years. Table 3 provides an overview of the average scores of the participating teams. Note that this corresponds to the median value of 2.45 and 2.97 (see Fig. 1). A significant difference was found between the two groups (t(13) = 2.53, p < 0.05). The average scores were 2.42 (SD = 0.44) and 2.95 (SD = 0.37) for groups 1 and 2, respectively. This indicates that the ART monitor is able to discriminate between the two groups.

**Table 3:** Average scores of participating teams

Group	Team	Average score
Group 1: Expected low-scoring group	1	1.87
	2	2.03
	3	2.08
	4	2.45
	5	2.70
	6	2.90
	7	2.93
Group 2: Expected high-scoring group	8	2.27
	9	2.68
	10	2.89
	11	2.95
	12	3.00
	13	3.08
	14	3.18
	15	3.52

#### Inter-rater reliability

Table 2 provides an overview of the inter-rater reliability of the items in the ART monitor, including the percentage of exact agreement and the percentage of agreement allowing a one-point difference in the case of a five point scale. Based on the exact agreement, six items (12%) scored above the threshold of 75%, and nineteen items (37%) scored above the threshold of 65%. When allowing a one-point difference, 30 items (59%) scored above the threshold of 75%, and 45 items (88%) scored above the threshold of 65%. This shows that when using a high standard, inter-rater reliability was weak; yet, the differences between the auditors were not that large since, when allowing for a one-point difference and a threshold of 65%, the agreement was strong.

### Finalizing the ART monitor

Based on the inter-rater reliability and content validity results, some changes were made to the ART monitor items. First, scoring options 2 and 4 were added to the sixteen items only allowing a score of 1, 3, or 5. This did not entail a change in the items. Second, three items (“Caseload”, “Rooming in”, and “Regional teams”) were removed because their content was already found in other items. This can be regarded as a small change in the instrument. Third, three items (“Leadership and pioneers in the team”, “(Mild) intellectual disabilities”, and “Attention to safety”) were added based on the feedback of the auditors and teams. Also, some criteria were added to existing items. The addition of new items can be considered a large change; however, the addition of new criteria is a small change. Fourth, items were clarified or specified. The description of a number of items was slightly rephrased or a definition was added for clarification. Mainly items that received a great deal of feedback and had a low inter-rater reliability were critically evaluated for possible semantic improvements to ensure the proper comprehension and scoring of the items. Also, for some items, a distinction was made between two settings, namely a clinical ward and a sheltered housing facility. Finally, the titles of five domains were rephrased; for example, the title of the domain “Team process” was rephrased into “Team culture and vision”, and some items were moved to other domains to make the structure of the instrument more coherent; for instance, items that focus on organizational elements of the model such as “Intake”, “Care coordination meeting”, “Systematic risk assessment”, and “Digital whiteboard”, were moved from the domain “Recovery-oriented care and support” to the domain “Organization of care”. The clarification, specification, and replacement of items did not result in a change in content. Additional File 2 comprises the English translation of the revised ART monitor.

## Discussion

The focus of this study was to evaluate the psychometric properties of the ART monitor. Content validity, construct validity, and inter-rater reliability of the instrument were investigated. First, regarding content validity, auditors and teams perceived the ART monitor as a useful instrument. Second, with regard to construct validity, a significant difference in scores on the ART monitor was found between the teams that were at the start of the implementation process (group 1) and teams that had been working with the ART model longer (group 2). Third, more than half the items (59%) had an inter-rater agreement of more than 75%, and the majority of the items (88%) had an inter-rater agreement of over 65%, when allowing for a one-point difference in scores. Items that scored low, in combination with feedback from auditors and teams, were revised. Most revisions were minor, simply involving scoring option additions, clarification and specification of items, and removal of redundancies. The addition of three items can be regarded as a larger change.

The approach in the current study was similar to that in the research focusing on the HIC monitor for acute psychiatric wards [25]. Scores on the ART monitor appeared to be less consistent when compared to the results of the HIC study, specifically the lower percentage of agreement and the larger number of revisions that were needed. An explanation could be that the settings in which the ART model is being implemented are more diverse than the HIC setting. ART settings range from closed long-stay wards to housing facilities; whereas HIC is being implemented in acute psychiatric wards, which are more comparable throughout the country. In addition, due to the length of admissions, the care provided by an ART team might be less structured compared to that provided at an HIC ward.

The ART model is a comprehensive and complex care model. Comparable to the fidelity measures Teague et al. [28] identified, the ART monitor includes items focusing on both the process and structural components of care and support. The content of the ART monitor is comparable to that of other instruments, such as the QuIRC and the ROPI used in long-term mental health care [17, 18]. The ART monitor and QuIRC include standards for disciplines, staff competencies, and housing. The QuIRC is a management tool; whereas the ROPI and the ART monitor are scored by independent auditors, thus providing a more holistic and independent evaluation of the quality of care. In contrast to the ROPI and QuIRC, the ART monitor is based on the ART model as a broadly shared vision developed in collaboration with many stakeholders. By breaking down the fundamental contents of the ART model into smaller parts (i.e., the individual items), teams have more concrete guidance for their implementation process. This makes the ART monitor a valuable instrument for providing a detailed overview of the situation within a team, assessed by independent auditors. For future research, including either the ROPI or QuIRC will be valuable for investigating the predictive validity of the ART monitor, in combination with outcomes on the service user level.

A strength of the current study is the set-up of the data collection, namely through a network of auditors, creating a Community of Practice [29]. This not only allowed for data collection, but it also created an opportunity for sharing knowledge and experiences, so that implementation of care according to the ART model was supported. Moreover, fruitful cooperation has been established for scientific evaluation of the ART monitor. Auditors and teams perceived the audits as inspiring, and in general, teams valued the feedback based on the audit results. This monitoring and feedback allowed them to critically reflect together on the care and support they provide, thereby contributing to the degree of compliance with the ART model within individual teams [11]. Points for improvements retrieved from the audits and subsequent discussions were adopted by teams in quality and policy documents. Teams that were at the start of their implementation process particularly benefited from participation in this study, because it allowed them to review their care process in a more structured manner than normal. By including these teams, it was ensured that the content of the ART monitor is clear for people with less experience with the ART model.

However, there are also limitations. First, only the scores of the ‘regular’ care professionals were included. In the development of the ART monitor, we aimed at a usable instrument for people with various backgrounds. Yet, in practice, this did not work out exactly as we anticipated. Limited knowledge of the structure of clinical records was indicated to be a barrier for auditors without the background of ‘regular’ care professionals. In addition, differences in scores could also have been the result of the limitations in the descriptions of the items in the ART monitor itself, which have undergone slight revisions based on this research. A third explanation could be that (family) peer workers and professionals with a ‘regular’ care background differ in their views on and expectations of recovery-oriented care, resulting in a different weighting of certain criteria of the ART monitor. In the current research, we were not able to look into this matter further due to the limited number of (family) peer worker auditors. Future research with a greater representation of (family) peer workers among the auditors could provide further insight on this issue. Also, lessons learned regarding the training of auditors are that we need to pay specific attention to scoring strategies and skills related to checking clinical records. We have learned that for some (family) peer workers more guidance is needed in the preparation and reporting of audits. Trust and an equal position among the other auditors are also important points of attention. In doing so, we will be able to include the perspectives of the whole triad (professional, peer worker, and family peer worker) in the audit process, which is considered very valuable during an audit. (Family) peer worker auditors are able to ask questions from a different angle and highlight possible blind spots of care workers that would strengthen the further implementation of the ART model in clinical practice. Second, the participating teams did not start the implementation of the ART model at precisely the same time. Although this would have been ideal for investigating construct validity, it was not possible for the current project. Since the research took place in a clinical setting in which the adoption of recovery-oriented care differs between teams, regardless of the starting date of the implementation of the ART model, a fixed point of comparison was not available. Nevertheless, we believe that this variability between teams gave us the opportunity to evaluate the ART monitor in a variety of settings when it comes to recovery-oriented care. This likely increased the clarity and usability of the instrument, including people with less experience with the ART model. Third, during the study, it became clear that several items of the ART monitor needed refinement. Special attention was paid to the items with a relatively low inter-rater reliability. A lack of scoring opportunities was perceived for the items with only three scoring options. For the remaining items with relatively low inter-rater reliability, the description of some criteria appeared insufficient. Refinements to these items were made, and further research will be needed to demonstrate whether the psychometric properties, especially the inter-rater reliability, have been further improved.

## Conclusion

This study focused on the evaluation of the psychometric properties of the ART monitor and further improvement of the instrument in close collaboration with Dutch mental health care organizations. The evaluation of the content validity, construct validity, and inter-rater reliability provided fruitful input for these goals. We concluded that the revised ART monitor is feasible and useful in mental health care practice. Continuous refinement and adaptation will be necessary as the field progresses and changes over time. Further evaluation of the psychometric properties of the revised ART monitor should be part of future research. This should include examining whether the inter-rater reliability of previously low-scoring items has improved as a result of the refinements. Furthermore, other psychometric properties, such as sensitivity to change and predictive validity, could be taken into account. In addition, future research should focus on the relationship between the degree of compliance with the ART model and outcomes such as quality of care and recovery of service users.

## Supplementary Information

**Additional file 1.** Supplementary information about the ART Model

**Additional file 2.** English translation of the ART monitor

## Data Availability

The revised ART monitor is available in Dutch on http://art-psy.nl/wp-content/uploads/2019/11/ART-Monitor-Oktober-2019-gevalideerd.pdf. The English translation of the ART monitor is included in Additional file 2. For questions regarding the data retrieved in this study, the reader can contact the first author (LZ).

## Abbreviations

ART: Active Recovery Triad
FACT: Flexible Assertive Community Treatment
HIC: High and Intensive Care
ICC: Ntraclass Correlation Coefficient
QuIRC: Quality Indicator for Rehabilitative Care
ROPI: Recovery Oriented Practices Index
SD: Standard Deviation
