# Supplementary material for: The Active Recovery Triad monitor: evaluation of a model fidelity scale for recovery-oriented care in long-term mental health care settings
Source: BMC Psychiatry. 2022 May 19;22:346. doi: 10.1186/s12888-022-03949-5 (PMC9118770; doi:10.1186/s12888-022-03949-5)
Supplement: Supplementary file 1 — Additional file 1. Supplementary information about the ART Model [file 12888_2022_3949_MOESM1_ESM.docx]

**Additional file 1**

**Article title:**

The Active Recovery Triad monitor: Evaluation of a model fidelity scale for recovery-oriented care in long-term mental health care settings

**Journal name:**

BMC Psychiatry

**Authors:**

Lieke Johanna Cornelia Zomer (Amsterdam UMC)

Lisette van der Meer (University of Groningen; Lentis Psychiatric Institute)

Jaap van Weeghel (Tranzo/Tilburg University; Phrenos)

Anne Laura van Melle (Amsterdam UMC; GGZ inGeest)

Henrica Cornelia Wilhelmina de Vet (Amsterdam UMC)

Martijn Kemper (GGZ inGeest)

Guy Antoine Marie Widdershoven (Amsterdam UMC)

Yolande Voskes (Amsterdam UMC; Tranzo/Tilburg University; GGz Breburg)

**Corresponding Author:**

Lieke Zomer [l.zomer@amsterdamumc.nl](mailto:l.zomer@amsterdamumc.nl)

**SUPPLEMENTARY INFORMATION ABOUT THE ART MODEL**

***The three key principles of the ART model***

The Active Recovery Triad (ART) model is an integrated care model for long-term mental health care, focusing on the recovery process of people with serious mental illnesses [1]. Central in the ART model are three key principles: Active, Recovery, and Triad. In order to improve recovery, an *Active* attitude of *all* people involved (including mental health workers, service users, family or significant others) is crucial. Clear boundaries with regard to the expected duration of care are important to encourage this active attitude [2]. In addition, service users must be actively involved in their own treatment and care plan as well as their (future) living environment. The second principle, *Recovery*, entails the focus on recovery of health (physical and mental health), recovery of personal identity, recovery of daily life, and recovery of community functioning [3]. Within the ART model, several (psycho-social) interventions can be initiated to stimulate the recovery process of service users. The third principle is the firm embedding of *the Triad* in all layers of care organization. This principle emphasizes the importance of the cooperation between service user, staff, and family or significant others at the individual, team, and organizational level. More specifically this entails involving service users and their significant others in decision making regarding care and support (individual level), working with peer workers and family peer workers within the team (team level), and involving service users and significant others in policy development and organizational changes (organizational level).

***Development of the ART model***

The iterative development process of the ART model included several invitational and expert meetings to jointly define the mission and vision, draw the outline of the model, and reflect upon the principles of the model [4]. Important topics addressed in a report on a national action plan for people with serious mental illnesses (e.g. self-management, lifestyle, intensive care, involvement of family, employment, and recovery) formed the basis of the key principles of the ART model [5]. Also evidence based practices for people with serious mental illnesses in long-term mental health care were examined, such as individual placement and support (IPS [6]), cognitive adaptation training (CAT [7-8]), and the wellness recovery action plan (WRAP [9]). In addition, international literature on care and support for people with serious mental illnesses, especially the work of Hellen Killaspy and colleagues, formed an important source of inspiration.

During the development process, the three principles Active, Recovery, and Triad were defined, including suggestions for recovery-oriented interventions to foster these principles in daily practice [1]. In addition, seven steps were defined to structure recovery-oriented care and support [4]. These steps include: 1) an intake meeting, 2) building a relationship of trust, 3) organize Care Planning Meetings, 4) seek for contact or restore contact with family and significant others, 5) introduce the concept of recovery, 6) define strengths, needs, and wishes of the service user, and 7) develop a treatment and rehabilitation plan in which personal recovery goals are central. Furthermore, preconditions for the care and support in this setting were developed, including team composition, competencies of care workers, cooperation with the regional network and outpatient care, safety, prevention of coercion, housing conditions, and implementation and evaluation of ART into clinical practice.

***Development of the model fidelity scale***

In order to support mental health care teams with implementing the ART model into practice and to follow the implementation processes by research, it was decided to develop a model fidelity scale for the ART model. Experiences with existing care models and model fidelity scales for other mental health settings, namely High and Intensive Care (HIC) and Flexible Assertive Community Treatment (FACT) provided a source of inspiration [10-11]. The model fidelity scale, upon which all contributors reached consensus, the ART monitor, consisted of 51 items divided into nine domains. The items and domains represented the core principles of the ART model, as defined based on scientific evidence and practical knowledge and experiences of relevant stakeholders in the field of long-term mental health care [4]. The structure of the ART monitor was as follows, listed by domain of the instrument:

- 1) **Team structure** included items regarding the team composition in terms of disciplines.

Items: 1. Caseload; 2. Team composition; 3. Peer worker and family peer worker; 4. Nurses; 5. Nurse practitioner; 6. Social workers/residential support worker; 7. Occupational therapist; 8. Psychiatrist; 9. Health care psychologist/behavioral specialist; 10. Extra disciplines.

- 2) **Team process** addressed items related to the competencies of care workers regarding e.g. attitude and how they collaborate.

Items: 11. Vision and working method; 12. Community participation; 13. Hospitality and presence; 14. Attitude of staff; 15. Active recovery; 16. Working in the triad.

- 3) **Recovery-oriented care and support** included items based on the seven steps of the ART model for structuring the care and support and working on recovery (the seven steps are explained above and in Zomer et al., 2020).

Items: 17. Intake; 18. Care coordination meeting (CCM); 19. Revitalize or build resource group; 20. Introduce recovery; 21. Needs, strengths and wishes; 22. Integrated treatment and recovery plan; 23. Recovery interventions at four levels; 24. Systematic risk assessment; 25. Early warning sign plan; 26. Digital whiteboard; 27. Rooming in; 28. Stepped care; 29. Recovery assessment.

- 4) **Other principles of recovery-oriented care and support** comprised items regarding the professional aspects of the long-term care setting, including knowledge of professional guidelines, diagnoses and medication.

Items: 30. Mental health care standards; 31. Somatic care; 32. Medication policy; 33. Dual diagnosis.

- 5) **Organization of care** included important preconditions for the care process, regarding e.g. admission and discharge, waiting list and consultation.

Items: 34. Cooperation with FACT and other outpatient care teams; 35. Admission and discharge; 36. Care process and consultation; 37. Waiting list; 38. Reachability; 39. Regional teams; 40. ART-improvement curve.

- 6) **Professionalization** focused on items regarding training, education and reflection of team members.

Items: 41. Reflection; 42. Training and education; 43. Knowledge of regional network; 44. Team spirit.

- 7) **Healing environment** addressed important preconditions regarding the housing of service users.

Items: 45. Healthy living environment; 46. Housing first; 47. Housing conditions.

- 8) **Safety** captured items related to expertise on and dealing with safety and safety management.

Items: 48. Safety management system; 49. Conflict management and personal safety; 50. Cooperation agreements on safety.

- 9) **Reduction of coercion** focused on the evaluation of coercive measures.

Items: 51. Evaluation of coercive measures.

For every item, scoring options on a scale from 1 to 5 were defined. Experts involved in the development of the HIC monitor and FACT monitor reviewed the items and scoring options on their feasibility. Furthermore, based on a first pilot of four audits the ART monitor was assessed and feedback was gathered on the relevance, comprehensiveness, and comprehensibility of the instrument. Small adaptations were made, including the correction of typing errors, clarifications of concepts, and small alterations in the items on housing conditions and the evaluation of coercive measures.

**REFERENCES**

[1] van Mierlo T, van der Meer L, Voskes Y, Berkvens B, Stavenuiter B, van Weeghel J. De kunst van ART. Werkboek Active Recovery Triad. Utrecht: De Tijdstroom; 2016

[2] Taylor Salisbury T, Killaspy H, King M. The relationship between deinstitutionalization and quality of care in longer-term psychiatric and social care facilities in Europe: A cross-sectional study. European Psychiatry, 2017;42:95-102. <https://doi.org/10.1016/j.eurpsy.2016.11.011>

[3] Dröes J, Plooy A. Herstelondersteunende zorg in Nederland: een vergelijking met Engelstalige literatuur. Tijdschrift voor Rehabilitatie. 2010;19:6–17.

[4] Zomer LJC, Van Der Meer L, Van Weeghel J, Widdershoven GAM, Van Mierlo T, Berkvens BS, et al. The Active Recovery Triad (ART) Model: a New Approach in Dutch Long-term Mental Health Care. Frontiers in Psychiatry. 2020;11:1169. <https://doi.org/10.3389/fpsyt.2020.592228>

[5] Projectgroep Plan van Aanpak EPA. Crossing the Bridge. Utrecht: Kenniscentrum Phrenos, 2014. Available online at: [www.kenniscentrumphrenos.nl/wpcontent/uploads/2014/10/Over-de-brug-PvA-EPA-september-2014.pdf](http://www.kenniscentrumphrenos.nl/wpcontent/uploads/2014/10/Over-de-brug-PvA-EPA-september-2014.pdf). [Accessed 7 December 2020].

[6] van Weeghel J, Bergmans C, Couwenbergh C, Michon H, de Winter L. Individual placement and support in the Netherlands: Past, present, and future directions. Psychiatric Rehabilitation Journal, 2019;43(1):24. <https://doi.org/10.1037/prj0000372>

[7] Stiekema AP, Quee PJ, Dethmers M, van den Heuvel ER, Redmeijer JE, Rietberg K, et al. Effectiveness and cost-effectiveness of cognitive adaptation training as a nursing intervention in long-term residential patients with severe mental illness: study protocol for a randomized controlled trial. Trials, 2015;16(1):49. <https://doi.org/10.1186/s13063-015-0566-8>

[8] Velligan DI, Mahurin RK, Eckert SL, Miller AL, BowThomas CC. Cognitive adaptation training: The use of compensatory strategies for inpatients and outpatients with schizophrenia. Schizophrenia Research, 1997;24(1-2):229-229. <https://doi.org/10.1016/S0920-9964(97)82662-X>

[9] Cook JA, Copeland ME, Jonikas JA, Hamilton MM, Razzano LA, Grey DD et al. Results of a randomized controlled trial of mental illness self-management using Wellness Recovery Action Planning. Schizophrenia bulletin, 2012;38(4):881-891. <https://doi.org/10.1093/schbul/sbr012>

[10] Nugter MA, Engelsbel F, Bähler M, Keet R, van Veldhuizen R. Outcomes of FLEXIBLE assertive community treatment (FACT) implementation: a prospective real life study. Community Mental Health Journal, 2016;52(8):898-907. <https://doi.org/10.1007/s10597-015-9831-2>

[11] van Melle AL, Voskes Y, de Vet HCW, van der Meijs J, Mulder CL, Widdershoven GAM. High and Intensive Care in Psychiatry: Validating the HIC Monitor as a Tool for Assessing the Quality of Psychiatric Intensive Care Units. Administration and Policy in Mental Health and Mental Health Services Research. 2019;46(1):34-43. <https://doi.org/10.1007/s10488-018-0890-x>
