# Supplementary material for: The Active Recovery Triad monitor: evaluation of a model fidelity scale for recovery-oriented care in long-term mental health care settings
Source: BMC Psychiatry. 2022 May 19;22:346. doi: 10.1186/s12888-022-03949-5 (PMC9118770; doi:10.1186/s12888-022-03949-5)
Supplement: Supplementary file 2 — Additional file 2. English translation of the ART monitor [file 12888_2022_3949_MOESM2_ESM.pdf]

ART Monitor

Active Recovery Triad Model Fidelity Scale

***!** Disclaimer: This concerns an English translation of the Dutch ART monitor. The validity and reliability of the Dutch ART monitor were investigated.*

Version October 2019

The ART Monitor is the intellectual property of the HIC & ART Foundation.

For more information about the application of the ART monitor, please email ART@vumc.nl or visit www.art-psy.nl

Instructions for completing the ART Monitor

ART Monitor layout based on two scenarios (ART workbook page 82):

- **Scenario 1.** The support team is responsible for the daily support; the (outpatient) treatment team is responsible for the treatment.
- **Scenario 2.** The support team and the treatment staff are integrated into one team.

! NOTE: for both scenarios, items are formulated separately in the ART Monitor.

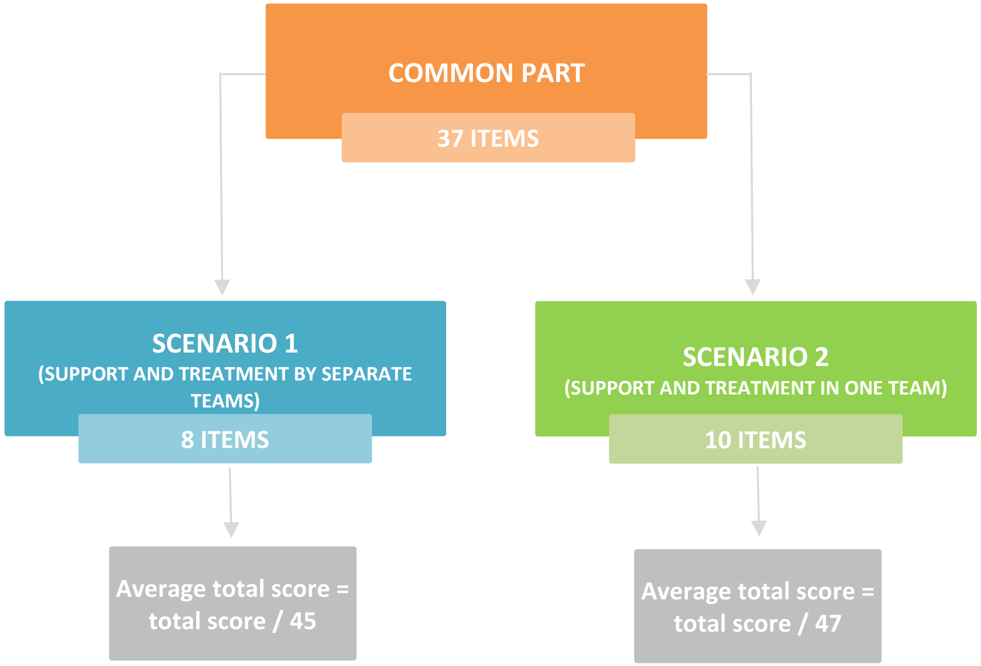

	Criterion	Score 1	2	3	4	5
**Domain 1: Recovery**						
Scenario 1 & 2	1. Active recovery<br>• Service users and significant others experience the team as recovery oriented.<br>• Not the problem behavior, but the basic needs of the service user are the primary basis of care and support. Each service user is systematically asked about their basic needs, strengths and wishes.<br>• The team actively demonstrates that recovery is possible for everyone. Service users are made aware of the possibilities of recovery.<br>• All team members show that the care and support are temporary; this is communicated with the service user and significant others in detail.	None of the criteria are met.	One of the criteria is met.	Two of the criteria are met.	Three of the criteria are met.	All of the criteria are met.

Scenario 1 & 2	2. Recovery interventions at four levels<br>Service users are actively motivated and make visible use of a wide range of recovery interventions, both inside and outside the organization (https://erkendeinterventiesggz.trimbos.nl/erkende-interventies). The service user's recovery needs are leading in the selection of an instrument. A demonstrable choice is made for recovery interventions aimed at:<br>• Recovery of health (mental and physical recovery).<br>• Recovery of identity (strengths, life story, personal goals, relationship, purpose, intimacy).<br>• Recovery of daily life (self-care, daily activities, finances).<br>• Recovery of community functioning (activation, work, education/training).	Service users cannot use recovery interventions.	Service users can use recovery interventions at one of the mentioned levels.	Service users can use recovery interventions at two of the mentioned levels. The service user's recovery needs are leading in the selection of an instrument.	Service users can use recovery interventions at three of the mentioned levels. The service user's recovery needs are leading in the selection of an instrument.	Service users can use recovery interventions at all levels. The service user's recovery needs are leading in the selection of an instrument.

Scenario 1 & 2	3. Community participation<br>The ART team is committed to community participation in the following way:<br>• With all service users, steps are taken towards social activation and improving community participation, adjusted to the individual level and the service user's wishes.<br>• The participation ladder is used for each service user, to gain insight into the level of community participation.<br>• The entire team is aware of and feels responsible for community participation.<br>• The team actively seeks opportunities for service users in the context of community participation outside the organization (e.g., social enterprises, community centers, walk-in facilities, associations, voluntary organizations, employers).<br>• The team is demonstrably in contact with municipalities in the context of enabling community participation.<br>• Rehabilitation methods are systematically used to promote community participation (e.g., IRB and the CARe model).	None of the criteria are met.	One or two of the criteria are met.	Three of the criteria are met.	Four or five of the criteria are met.	All of the criteria are met.

Scenario 1 & 2	4. Knowledge of the regional network<br>• The team is focused on the external environment.<br>• The team knows about and pays attention to social services in the region and seeks for opportunities and expertise within the regional network in terms of recovery oriented care for people with severe mental illness.<br>• The team is aware of the vision and working method of relevant partners in the regional network, and these relevant partners are aware of the team's vision and working method. They try to find common ground and align with one another.	None of the criteria are met.	One of the criteria is met.	-	Two of the criteria are met.	All of the criteria are met.
Scenario 1 & 2	5. Mental health care standards<br>• The team demonstrates to work with diagnostic-specific guidelines and mental health care standards at the individual service user level.<br>• The team is familiar with SMI (Serious Mental Illness) specific guidelines (Dutch: generieke module EPA) and actively puts it into practice.<br>• The team has a method of keeping its knowledge of mental health care standards up-to-date (for example, an attention officer who maintains their current knowledge of mental health care standards and regularly gives feedback about this to the rest of the team).	None of the criteria are met.	One of the criteria is met.	-	Two of the criteria are met.	All of the criteria are met.

Scenario 1 & 2	6. Somatic care<br>• The team has an identifying role in general medical care, hospital care and dental care and ensures that service users comply with agreements and appointments in these areas.<br>• The team plays an active role in somatic care and healthy lifestyles and provides service users with lifestyle advice.<br>• The team demonstrates that they screen the physical health of all service users annually (physical examination, metabolic screening and lab tests).	None of the criteria are met.	One of the criteria is met.	-	Two of the criteria are met.	All of the criteria are met.

Scenario 1 & 2	7. Medication policy<br>There is a protocol-based medication policy that is based on the most current insights/guidelines. This medication policy meets the following requirements:<br>• Agreements on medication have been reached based on shared decision-making with the service user and significant others.<br>• Specific attention is paid to the prevention and/or reduction of polypharmacy.<br>• Medication agreements are clearly described and are structurally evaluated in the triad.<br>• There is a structural alignment between the treatment practitioner and the team (either multidisciplinary consultation in the team or coordinated with the general practitioner and/or external treatment team).<br>• The team is committed to providing support, psycho-education and health education regarding medication use and its side effects.<br>• The medication policy is based on the latest insights into medication and medication remediation, and the team knows the nursing guidelines for medication use.	None of the criteria are met.	One or two of the criteria are met.	Three of the criteria are met.	Four or five of the criteria are met.	All of the criteria are met.

	Criterion	Score 1	2	3	4	5
**Domain 2: The triad**						
Scenario 1 & 2	8. Working in the triad: service user level<br>• Each service user is actively asked who could help them in their recovery process. Significant others are not just family members, but could also be friends, colleagues, neighbors, etc.<br>• Significant others are actively approached. The team is flexible in this and coordinates meetings in agreement with the significant others.<br>• Significant others are actively involved in making decisions (shared decision-making).<br>• Service users and significant others are actively supported in the recovery process.<br>• If necessary, home visits to the significant others take place as part of restoring relationships.<br>• Systemic family therapy or other meetings can be conducted with the aim of restoring disturbed relationships.	None of the criteria are met.	One or two of the criteria are met.	Three of the criteria are met.	Four or five of the criteria are met.	All of the criteria are met.

Scenario 1 & 2	9. Working in the triad: team level and organizational level<br>• The team is familiar with guidelines for working with and supporting significant others (Dutch: generieke module Samenwerking en ondersteuning naasten).<br>• At the team level, service users and significant others are involved in decision making and changes.<br>• The team regularly asks for feedback from the service users. The team actively uses this feedback to improve their actions or procedures.<br>• The team regularly asks for feedback from significant others. The team actively uses this feedback to improve the actions or procedures (Dutch: for example, using the Quickscan GGZ 'Werken met familie').<br>• Service users and significant others are involved in policy-making and changes at the organizational level.	None of the criteria are met.	One of the criteria is met.	Two or three of the criteria are met.	Four of the criteria are met.	All of the criteria are met.

Criterion		Score 1	2	3	4	5
**Domain 3: Organization of care**						
Scenario 1 & 2	10. Intake<br>• During the intake process, relatable questions are used about the service user's life story and wishes for recovery.<br>• The service user and significant others receive information and explanation about the team's vision and working method.<br>• The team and the service user reflect upon previous treatment and support, and discuss what should be different in the current process.<br>• The service user and significant others can actively participate in the decision-making about the steps to be taken for treatment and support.<br>• The service user receives adequate information about the team members, and the team actively acts on the service user's preference for a personal key worker.	None of the criteria are met.	One of the criteria is met.	Two or three of the criteria are met.	Four of the criteria are met.	All of the criteria are met.

Scenario 1 & 2	11. Personal recovery plan<br>Service users are encouraged to develop a personal recovery plan (whether or not using WRAP). This recovery plan focuses on recovery wishes and the service user's personal profile. Service users always have access to their recovery plan. Service users are encouraged to include the following aspects in their recovery plan:<br>• Self-formulated recovery goals based on their recovery needs and wishes.<br>• Integration of treatment into the personal recovery plan.<br>• What kind of personal support, treatment, activities, participation coaching and residential support the service user needs to support the recovery wishes/goals.<br>• Within the goals it is clear who does what (responsibilities, roles and division of tasks), and the team reports on these goals with the service user.<br>• The recovery plan is evaluated every six months within the triad (preferably in ZAG); adjustments can be made to the plan if necessary.	None of the criteria are met, or the service user does not always have access to the recovery plan.	One or two of the criteria are met, but the workers are still preparing the recovery plan. The service user always has access to the recovery plan.	Three of the criteria are met, but the workers are still preparing the recovery plan. The service user always has access to the recovery plan.	Four of the criteria are met, but the workers are still preparing the recovery plan. Or three of the criteria are met, but the recovery plan (primarily, at least the recovery goals) is developed by the service user.	Four or all of the criteria are met and the recovery plan (primarily, at least the recovery goals) is developed by the service user.

Scenario 1 & 2	12. Care coordination meeting<br>Care coordination meetings take place regularly. These are linked to the evaluation of the recovery plan. The treatment and recovery goals are developed and/or adjusted in the these meetings. The care coordination meeting meets the following criteria:<br>• In any case, the service user, the treatment practitioner, family and/or other significant others are present. At the service user's invitation, other disciplines and/or persons can participate.<br>• The first care coordination meeting takes place within the week of admission. During this meeting, agreements are made about the intended duration of the treatment and support. The personal recovery plan is leading (in the absence of a personal recovery plan, the service user is actively encouraged to develop it).<br>• The second care coordination meeting takes place after 6-8 weeks.<br>• The remaining care coordination meetings take place on indication, but at least twice a year.<br>• When transferring care, a care coordination meeting takes place within the triad, with the party taking over the service user's care.	None of the criteria are met.	One of the criteria is met.	Two or three of the criteria are met.	Four of the criteria are met.	All of the criteria are met.

Scenario 1 & 2	13. Systematic risk assessment<br>The team demonstrates to work with instruments assessing:<br>• Acting-out behavior/aggression<br>• Crisis<br>• Suicidal risks	No risk assessment are used.	A risk assessment instrument is used on one of the subjects mentioned.	A risk assessment instrument is used on two of the subjects mentioned.	A risk assessment instrument is used on three of the subjects mentioned.	Risk assessment is carried out on all subjects, and this is integrated in the service user's care and support.
Scenario 1 & 2	14. Early warning sign plan<br>• All service users have an up-to-date early warning sign plan.<br>• The plan is written from the perspective of the service user.<br>• The early warning sign plan is developed and evaluated in the triad.<br>• The early warning sign plan is evaluated and adjusted at least once a year and after each crisis or relapse.<br>• The early warning sign plan is integrated into the service user's daily care and support.	None of the criteria are met.	One of the criteria is met.	Two or three of the criteria are met.	Four of the criteria are met.	All of the criteria are met.
Scenario 1 & 2	15. Digital whiteboard meeting<br>The digital whiteboard meeting meets the following criteria:<br>• The meeting follows a fixed structure.<br>• The team member's responsibilities are made clear during the meeting.<br>• The overview used during the meeting is up to date. After each meeting, changes are made, and they are transparent to everyone.<br>• The meeting results are leading for crisis prevention, the use of interventions, making additional arrangements, the scaling-up of care and 'shared caseload' where necessary.<br>• The team uses an digital overview and the board is linked to the client files.	The team meets none of the criteria.	The team meets one of the criteria set.	The team meets two or three of the criteria.	The team meets four of the criteria.	The team meets all the criteria.

Scenario 1 & 2	16. Stepped care<br>The following options are possible to intensify care:<br>• The team/personal key worker can recognize early warning signs of a crisis, and the service user is discussed during the digital whiteboard meeting. Agreements have been made in the team and with service users based on their early warning sign plan (criteria for stepped care).<br>• Intensify care to 1-on-1 support is possible in the service user's own environment.<br>• The schedule and the formation take into account the possible intensification of care.<br>• The option of involving significant others when the care is intensified is clearly described and is used when possible.<br>• There are structural agreements with acute wards in the region for temporary admissions.	None of the criteria are met.	One of the criteria is met.	Two or three of the criteria are met.	Four of the criteria are met.	All of the criteria are met.
Scenario 1 & 2	17. Recovery assessment<br>Once a year, a recovery scale is assessed with all service users (this can be part of the routine outcome monitoring). Results of this recovery scale are used in the daily treatment and support and of service users.	No recovery scale is used.	-	A recovery scale is used once a year for all service users. The results of this recovery scale are not used.	-	A recovery scale is used once a year for all service users. The results are used to fine-tune/adjust the service user's daily treatment and support.

Scenario 1 & 2	18. Admission and discharge<br>• The organization's policy is focused on service user admission and discharge, with clear criteria.<br>• Admissions are focused on the wishes and personal process of the service user.<br>• There is no waiting list to start care.<br>• Collaboration with partners in the region is actively sought to enable the process of admission and discharge.	None of the criteria are met.	One of the criteria is met.	Two of the criteria are met.	Three of the criteria are met.	All of the criteria are met.
Scenario 1 & 2	19. Care process and consultation<br>• Internal consultation can be sought when a team, the service user, and their significant others reach a stagnation point in the recovery.<br>• At least after three years, an internal or external consultation will be conducted for each service user to evaluate treatment, care and support.<br>• The consultation leads to new recovery goals in the recovery plan aiming at rediscovering the journey towards recovery.	None of the criteria are met.	One of the criteria is met.	-	Two of the criteria are met.	All of the criteria are met.

	Criterion	Score 1	2	3	4	5
**Domain 4: Team culture and vision**						
Scenario 1 & 2	20. Vision and working method<br>The team works with a clearly described vision and working method, in line with the principles of the ART model. All team members are well aware of this vision, apply it into daily practice, and communicate with service users, significant others, internal and external partners.	No vision and/or working method has been described.	The team is in the process of developing vision and working method.	The team has a clearly described vision and working method in line with the principles of the ART model, but the awareness and use of this vision depends on the individual employee.	The team has a clearly described vision and working method, in line with the principles of the ART model. All team members are well aware of this vision, but applying it into daily practice and communicating about it with service users, significant others, internal and external partners depends on the individual employee.	The team has a clearly described vision and working method, in line with the principles of the ART model. All team members are well aware of this vision, apply it into daily practice and communicate about it with service users, significant others, internal and external partners.

Scenario 1 & 2	21. Attitude of staff<br>The team approach meets the following criteria:<br>• All team members approach service users in a consistent manner and based on an unconditional relationship and equality.<br>• The team recognizes the importance of constant and sincere contact with service users and applies the principles of methods such as the 'first five minutes of admission' and the presence approach.<br>• Significant others are always hospitably welcomed. They feel welcome.<br>• All team members treat the service user's personal environment with respect. They are aware that they are visiting the service users (for example: knocking, waiting for permission to enter).<br>• The team adheres to all agreements that are made with service users and significant others.	None of the criteria are met.	One of the criteria is met.	Two or three of the criteria are met.	Four of the criteria are met.	All of the criteria are met.

Scenario 1	22. Presence and reachability: Scenario 1 (support and treatment by separate teams)<br>• Service users and significant others feel that team members are present.<br>• Workers report in the common group area; there is no closed office.<br>• During the day, there is always at least one worker available for service users and significant others.<br>• If necessary (crisis), the team is available in at least 20 minutes.<br>• In case of a crisis outside office hours, there are protocolled agreements with acute wards.<br>• Service users and significant others are actively informed about the team's availability in case of questions or in case of an emergency.	None of the criteria are met.	One or two of the criteria are met.	Three of the criteria are met.	Four or five of the criteria are met.	All of the criteria are met.
Scenario 2	22. Presence and reachability: Scenario 2 (support and treatment by one team)<br>• Service users and significant others feel that team members are present.<br>• Workers report in the common group area; there is no closed office.<br>• During the day, there are always at least two employees available for the service users and significant others.<br>• If necessary (crisis), a treatment practitioner is available in at least 20 minutes.<br>• Service users and significant others are actively informed about the team's availability for questions or in case of emergency.	None of the criteria are met.	One of the criteria is met.	Two or three of the criteria are met.	Four of the criteria are met.	All of the criteria are met.

Scenario 1 & 2	23. Collaboration of support team and treatment staff<br>• The support team and (outpatient) treatment staff experience a pleasant mutual collaboration.<br>• The contact between treatment staff and the support team is easy, and they can quickly connect and make decisions when needed.<br>• Structural work arrangements have been made between the support team and the treatment staff regarding collaboration (moments of contact, when to involve one another in care and support, etc.).	None of the criteria are met.	One of the criteria is met.	-	Two of the criteria are met.	All of the criteria are met.
Scenario 1 & 2	24. Team spirit<br>The team scores positively on the following items:<br>• All team members agree that the atmosphere in the team is open and pleasant.<br>• All team members agree on the team's working methods and objectives; they share the same views.<br>• There is mutual support and commitment. Team members feel that they can trust and depend on each other.<br>• Team members give each other positive and constructive feedback. Team members are not afraid to confront others about their actions.	None of the criteria are met.	One of the criteria is met.	Two of the criteria are met.	Three of the criteria are met.	All of the criteria are met.

Scenario 1 & 2	25. Reflection<br>• Team members are able to critically reflect upon their own actions and take into account feedback from others to improve their actions.<br>• The team reflects on their actions during team meetings, case reflection meetings, and moments in daily care.<br>• Where necessary, team members consciously use their (personal) experiences in their professional context.<br>• Each team member participates in (group) supervision (such as moral case deliberation) for at least 6x2 hours a year. The themes discussed during the these meetings are tailored to the needs of the team.	None of the criteria are met.	One of the criteria is met.	Two of the criteria are met.	Three of the criteria are met.	All of the criteria are met.

Scenario 1 & 2	26. Innovation and improvement<br>• There is a high level of innovation; the team is brimming with ideas and has the decisiveness to implement them.<br>• The organization supports the team in developing new ideas (workers receive time and space for this and/or project leaders/staff members are employed to support the team in this process).<br>• The team members and team leaders regularly monitor the quality of care. For example, they regularly review the ART monitor. The results are used to improve care and support. For example, the Plan-Do-Check-Act cycle is used for improvement.<br>• There is a structural exchange of knowledge and experiences between teams within and outside the organization.<br>• National developments related to recovery oriented care and new interventions are actively followed by the team and shared in the team and the organization.	None of the criteria are met.	One of the criteria is met.	Two or three of the criteria are met.	Four of the criteria are met.	All of the criteria are met.

Scenario 1 & 2	27. Leadership and pioneers in the team<br>• The team leader or manager is involved in the team and is available for the team members.<br>• The team leaders or manager lead from a positive fundamental attitude. This person can motivate and enthuse the team.<br>• Pioneers within the team actively keep track of the ART developments and get the rest of the team involved.<br>• Team members have the space to make decisions themselves.<br>• The team is encouraged to celebrate (even small) successes, at the team and service user level.	None of the criteria are met.	One of the criteria is met.	Two or three of the criteria are met.	Four of the criteria are met.	All of the criteria are met.

Criterion		Score 1	2	3	4	5
**Domain 5: Professionalization**						
Scenario 1 & 2	28. Training and education<br>There is sufficient attention paid to the development and expertise of team members:<br>• All employees have a personal development/training plan.<br>• All employees receive a minimum of four training or education sessions (half-day) each year.<br>• There are various training opportunities in terms of content (recovery support and rehabilitation, cooperation in the triad and resource groups, making contact, communication, treatment, attitude, peer work, somatic care).<br>• Employees can reflect on their training needs. These needs are discussed and evaluated once a year.	None of the criteria are met.	One of the criteria is met.	Two of the criteria are met.	Three of the criteria are met.	All of the criteria are met.

Scenario 1 & 2	29. Dual diagnosis<br>• Within the team, attention is paid to the improvement of expertise in the field of dual diagnosis.<br>• The entire team is structurally trained in motivational interviewing.<br>• In case of a dual diagnosis, addiction treatment is integrated. There is a combined focus on (the interactions between) psychiatric and addiction problems according to 'Integrated Dual Disorder Treatment' (IDDT).<br>• An instrument is used to assess addiction problems (for example MATE, Subjective Abstinence Scale, Objective Abstinence Scale, Clinical Withdrawal Scale).<br>• The team includes an employee with experience in the addiction sector, or the team has warm connections with addiction care to import this knowledge and expertise when needed.	None of the criteria are met.	One of the criteria is met.	Two or three of the criteria are met.	Four of the criteria are met.	All of the criteria are met.

Scenario 1 & 2	30. (Mild) intellectual disability<br>• The team pays attention to the improvement of expertise in the field of intellectual disabilities.<br>• An instrument is used to screen for a (mild) intellectual disability (for example: SCIL).<br>• In case of (mild) intellectual disability, the team adjusts the treatment and support accordingly.<br>• The team includes an employee with experience working with intellectual disabilities, or the team has connections to specialized organizations on intellectual disability to import this knowledge and expertise when needed.	None of the criteria are met.	One of the criteria is met.	Two of the criteria are met.	Three of the criteria are met.	All of the criteria are met.

Criterion		Score 1	2	3	4	5
**Domain 6: Healing environment**						
Scenario 1 & 2	31. Healthy living environment<br>• The physical environment is designed to promote the well-being of service users, significant others and team members (sufficient daylight, fresh air, plants, use of natural materials).<br>• Attention is paid to a healthy living environment. There is a joint responsibility to keep the common (outdoor) areas clean and tidy.<br>• The team coaches service users to maintain a healthy living environment.<br>• Workers set a good example for lifestyle themes such as smoking and healthy nutrition.	None of the criteria are met.	One of the criteria is met.	Two of the criteria are met.	Three of the criteria are met.	All of the criteria are met.

Scenario 1 & 2	32. Housing preferences<br>• At the start of care, every service user is actively asked about their wishes and preferences regarding their housing.<br>• Based on the service user's individual situation and wishes, a well-considered choice is made within the triad about the best living situation for the service user.<br>• During the service user's recovery process, the team will question the service user about wishes and evaluate whether the current housing matches these wishes. The team tries to do everything possible to respond to this (for example, moving someone to another place of residence within or outside the organization if this is more appropriate).	None of the criteria are met.	One of the criteria is met.	-	Two of the criteria are met.	All of the criteria are met.
Scenario 1 & 2	33. Housing conditions<br>• Private living room, bedroom and sanitary facilities.<br>• Cooking facilities are available if the service user prefers it.<br>• All service users have their own key.<br>• There is an outdoor area or garden.<br>• Rooms can be furnished and decorated according to personal wishes. The team thinks along with this.<br>• Significant others can stay over during the night.<br>• There is a common group area.	None of the criteria are met.	One or two of the criteria are met.	Three or four of the criteria are met.	Five or six of the criteria are met.	All of the criteria are met.

	Criterion	Score 1	2	3	4	5
**Domain 7: Safety and coercion**						
Scenario 1 & 2	34. Safety management system<br>Structural reports, analyses and improvement actions are anchored in the Safety management system.	There is no safety management system.	Incident reporting depends on individual workers.	There is a safety management system. Structural reporting takes place, but there are no analyses and improvement actions.	There is a safety management system that is used for structural reporting. Based on the reports, analyses are carried out for safety quality improvement, but the team does not always use the results in daily practice.	There is a safety management system that is used for structural reporting. Based on the reporting, analyses are carried out for safety quality improvement, and the team actively uses the results to improve daily practice.
Scenario 1 & 2	35. Conflict management and personal safety<br>There is an organizational policy regarding conflict management and personal safety. Best practices such as the Mat, negotiation techniques and de-escalation techniques are used. Staff receive annual training on these themes.	There is no policy regarding conflict management and personal safety, and no best practices are used.	There is occasional training in conflict management and personal safety; best practices are not used in daily practice.	There is occasional training in conflict management and personal safety; the application of best practices in daily practice depends on the individual worker.	The team is trained annually in conflict management and personal safety; the application of best practices in daily practice depends on the individual worker.	There is a clear policy regarding conflict management and personal safety. The team is trained annually, and the entire team knows and applies best practices in daily practice.

Scenario 1 & 2	36. Cooperation agreements on safety<br>There are demonstrable cooperation agreements on safety with key partners (for example, police, security, referring partners, neighbors). These agreements include how people collaborate in a crisis or during incidents and how they ensure a safe environment. All team members are familiar with these agreements, and the agreements are applied in practice.	There are no agreements with other organizations on safety.	There is uncertainty within the team about agreements that have been made.	Agreements have been made demonstrably. Not all team members are aware of this.	Agreements have been made, team members know what has been agreed and know where to check or search for the content of these agreements.	Agreements have been made that are known to all team members and are applied in practice.
Scenario 1 & 2	37. Attention to safety<br>• Team members, service users and significant others experience a safe environment.<br>• After an incident, follow-up care is provided to service users, tailored to their needs.<br>• After an incident, follow-up care is provided to team members, tailored to their needs.<br>• After an incident, the follow-up care will be provided to the significant others, tailored to their needs.<br>• Safety is a subject during meetings in the triad.	None of the criteria are met.	One of the criteria is met.	Two or three of the criteria are met.	Four of the criteria are met.	All of the criteria are met.

Scenario 1 & 2	38. Evaluation of coercive measures<br>Coercive measures are used with extreme caution, and the team has an active policy to reduce coercion. The team meets the following criteria:<br>• A service user's early warning sign plan is leading in the application of coercive measures.<br>• All coercive measures are subsequently evaluated with the service user.<br>• The significant others are involved in the evaluation of coercive measures.<br>• The team uses the evaluation as a learning moment to structurally evaluate and adapt the course of action.<br>*(Coercive measures include seclusion, isolation, fixation, administration of liquids/food, administration of medication, restriction of freedom outside the ward or facility (e.g., the service user cannot go outside without supervision), limitation of communication (e.g., taking away a telephone) and all other restrictions that prevent someone from doing or not doing what they would do, within the rules of the law.)*	None of the criteria are met.	One of the criteria is met.	Two of the criteria are met.	Three of the criteria are met.	No coercive measures are used or all the criteria are met.

	Criterion	Score 1	2	3	4	5
	**Domain 8: Team structure**<br>*(long-term absentees and trainees are not included in the calculation)*					
Scenario 1	39. Team composition<br>The requirements of the team are:<br>• No more than 20% of contracts are contracts of less than 28 hours.<br>• No more than 20% employees have a temporary contract.<br>• The team is steady but active, with no more than 50% of the employees working in the team for more than 5 years.<br>• The team includes a mix of work experience, both in outpatient care and clinical care.	None of the criteria are met.	One of the criteria is met.	Two of the criteria are met.	Three of the criteria are met.	All of the criteria are met.
Scenario 1	40. Nurses and social workers<br>• There are at least 4.0 fte nurses (with their own caseload) available per 25 service users.<br>• There are at least 4.0 fte social workers (with their own caseload) available per 25 service users.<br>• At least 40% of the nurses and social workers have higher education (meaning: university of applied sciences of higher).	None of the criteria are met.	One of the criteria is met.	-	Two of the criteria are met.	All of the criteria are met.

Scenario 1	41. Peer worker<br>The team includes a minimum of 0.5 fte peer worker per 25 service users. This person works in addition to the daily formation, gives solicited and unsolicited critical advice to the team from the service user perspective, helps service users in their recovery by, among other things, the initiation of recovery interventions.	There is no peer worker available for the team, or they are not deployed.	The team deploys peer worker(s), who are available within the organization, on indication.	The team employs 0.24 fte or less peer worker(s), based on 25 service users.	The team employs 0.25 of 0.49 fte peer worker(s), based on 25 service users.	The team employs 0.5 fte or more peer worker(s), based on 25 service users.
Scenario 1	42. Family peer worker<br>The team includes a minimum of 0.5 fte family peer worker per 25 service users. This person works in addition to the daily formation, providing solicited and unsolicited critical advice to the team from the family perspective and is a focal point for family and significant others.	There is no family peer worker available for the team, or they are not deployed.	The team deploys family peer worker(s), who are available within the organization, on indication.	The team employs 0.24 fte or less family peer worker(s), based on 25 service users.	The team employs 0.25 of 0.49 fte family peer worker(s), based on 25 service users.	The team employs 0.5 fte or more family peer worker(s), based on 25 service users.
Scenario 1	43. Activity worker, participation coach and job coach<br>There is at least 1.0 fte activity worker, participation coach and job coach staff available for the team. These disciplines support the development and achievement of personal goals in daytime activities, work and learning.	There is 0,24 fte or less activity worker, participation coach and job coach staff available.	There is 0.25 to 0.49 fte activity worker, participation coach and job coach staff available.	There is 0.5 to 0.74 fte activity worker, participation coach and job coach staff available.	There is 0.75 to 0.99 fte activity worker, participation coach and job coach staff available.	There is 1.0 fte or more activity worker, participation coach and job coach staff available.
Scenario 1	44. Nurse practitioner<br>There is at least 0.5 fte nurse practitioner available per 25 service users. This nurse practitioner has a crucial role in the team (coach, treatment, integrated care).	There is 0.19 or less fte nurse practitioner available per 25 service users.	There is 0.2 to 0.29 fte nurse practitioner available per 25 service users.	There is 0.3 to 0.39 fte nurse practitioner available per 25 service users.	There is 0.4 to 0.49 fte nurse practitioner available per 25 service users.	There is 0.5 fte or more nurse practitioner available per 25 service users.

Scenario 1	45. Extra disciplines<br>The following disciplines are actively deployed when service users experience problems in one of these areas:<br>• Community worker<br>• Somatic specialist<br>• Occupational therapist<br>• Addiction expert<br>• Clergyman/spiritual worker<br>• Legal consultant/client confidant<br>• Geriatric expert<br>• Guardian/budget management	None or one of the disciplines is available to all service users.	Two or three of the disciplines are available for all service users.	Four or five disciplines are available for all service users.	Six or seven disciplines are available for all service users.	Eight disciplines are available for all service users.

	Criterion	Score 1	2	3	4	5
	**Domain 8: Team structure** *(long-term absentees and trainees are not included in the calculation)*					
Scenario 2	39. Team composition<br>The team requirements are:<br>• No more than 20% of contracts are contracts of less than 28 hours.<br>• No more than 20% of the employees have a temporary contract.<br>• The team is steady but active; no more than 50% of the employees have been working in the team for more than 5 years.<br>• The team includes a mix of work experiences, both in outpatient care clinical care.	None of the criteria are met.	One of the criteria is met.	Two of the criteria are met.	Three of the criteria are met.	All of the criteria are met.
Scenario 2	40. Nurses and social workers<br>• There are at least 4.0 fte nurses (with their own caseload) available per 25 service users.<br>• There are at least 4.0 fte social workers (with their own caseload) available per 25 service users.<br>• At least 40% of the nurses and social workers have higher education (meaning: university of applied sciences of higher).	None of the criteria are met.	One of the criteria is met.	-	Two of the criteria are met.	All of the criteria are met.

Scenario 2	41. Peer worker<br>The team includes a minimum of 0.5 fte peer worker per 25 service users. This person works in addition to the daily formation, gives solicited and unsolicited critical advice to the team from the service user perspective, helps service users in their recovery by, among other things, the initiation of recovery interventions.	There is no peer worker available for the team, or they are not deployed.	The team deploys peer worker(s), who are available within the organization, on indication.	The team employs 0.24 fte or less peer worker(s), based on 25 service users.	The team employs 0.25 of 0.49 fte peer worker(s), based on 25 service users.	The team employs 0.5 fte or more peer worker(s), based on 25 service users.
Scenario 2	42. Family peer worker<br>The team includes a minimum of 0.5 fte family peer worker per 25 service users. This person works in addition to the daily formation, providing solicited and unsolicited critical advice to the team from the family perspective and is a focal point for family and significant others.	There is no family peer worker available for the team, or they are not deployed.	The team deploys family peer worker(s), who are available within the organization, on indication.	The team employs 0.24 fte or less family peer worker(s), based on 25 service users.	The team employs 0.25 of 0.49 fte family peer worker(s), based on 25 service users.	The team employs 0.5 fte or more family peer worker(s), based on 25 service users.
Scenario 2	43. Activity worker, participation coach and job coach<br>There is at least 1.0 fte activity worker, participation coach and job coach staff available for the team. These disciplines support the development and achievement of personal goals in daytime activities, work and learning.	There is 0,24 fte or less activity worker, participation coach and job coach staff available.	There is 0.25 to 0.49 fte activity worker, participation coach and job coach staff available.	There is 0.5 to 0.74 fte activity worker, participation coach and job coach staff available.	There is 0.75 to 0.99 fte activity worker, participation coach and job coach staff available.	There is 1.0 fte or more activity worker, participation coach and job coach staff available.
Scenario 2	44. Nurse practitioner<br>There is at least 0.5 fte nurse practitioner available per 25 service users. This nurse practitioner has a crucial role in the team (coach, treatment, integrated care).	There is 0.19 or less fte nurse practitioner available per 25 service users.	There is 0.2 to 0.29 fte nurse practitioner available per 25 service users.	There is 0.3 to 0.39 fte nurse practitioner available per 25 service users.	There is 0.4 to 0.49 fte nurse practitioner available per 25 service users.	There is 0.5 fte or more nurse practitioner available per 25 service users.

Scenario 2	45. Psychiatrist<br>The psychiatrist meets the following criteria:<br>• There is at least 1 fte psychiatrist available per 100 service users.<br>• A resident psychiatrist (or other coordinating treatment practitioner) supervises the admission process and is responsible for the diagnosis and treatment.<br>• The psychiatrist is physically present at the location at least two days a week.<br>• The psychiatrist is easily accessible to service users and significant others.	The psychiatrist does not meet any of the criteria, or is not present at all.	The psychiatrist meets one of the criteria.	The psychiatrist meets two of the criteria.	The psychiatrist meets three of the criteria.	The psychiatrist meets all the criteria.
Scenario 2	46. Health care psychologist/behavioral specialist<br>The health care psychologist/behavioral specialist meets the following criteria:<br>• There is at least 1 fte health care psychologist/behavioral specialist per 100 service users.<br>• There is a resident health care psychologist/behavioral specialist who is responsible for or can be consulted during the admission process and for the diagnosis and treatment.<br>• The health care psychologist/behavioral specialist is physically present on the location at least three days a week.<br>• The health care psychologist/behavioral specialist is easily accessible to service users and the significant others.<br>• The health care psychologist/behavioral specialist is specialized in the treatment of service users with cognitive limitations.	The health care psychologist/ behavioral specialist does not meet any of the criteria, or is not present at all.	The health care psychologist/ behavioral specialist meets one of the criteria.	The health care psychologist/ behavioral specialist meets two or three of the criteria.	The health care psychologist/ behavioral specialist meets four of the criteria.	The health care psychologist/ behavioral specialist meets all criteria.

Scenario 2	47. Extra disciplines<br>The following disciplines are actively used when service users experience problems in one of these areas:<br>• Community worker<br>• Somatic specialist<br>• Occupational therapist<br>• Addiction expert<br>• Clergyman/spiritual worker<br>• Legal consultant/client confidant<br>• Geriatric expert<br>• Guardian/budget management	None or one of the disciplines is available to all service users.	Two or three disciplines are available for all service users.	Four or five disciplines are available for all service users.	Six or seven disciplines are available for all service users.	Eight disciplines are available for all service users.
